# Effects of Formula Product Injection on Hatching Parameters, Small Intestinal Development and Ileum Histology in Breeder Chicken Eggs

**DOI:** 10.1002/vms3.70153

**Published:** 2025-01-10

**Authors:** Oğuzhan Eray, Gökhan Filik

**Affiliations:** ^1^ Biotechnology Research Centre Central Research Institute for Field Crops, Research and Technology Development Campus, Yenimahalle Ankara Türkiye; ^2^ Department of Agricultural Biotechnology, Faculty of Agriculture University of Kırşehir Ahi Evran Kırşehir Türkiye

**Keywords:** chick quality, hatching, ileum histology, in ovo feeding, small intestinal development

## Abstract

**Background and Objective:**

This study aimed to determine the effects of in ovo formula product injection on hatching parameters, chick quality, small intestinal development and ileum histology of breeder hen eggs.

**Methods:**

A total of 400 fertilised eggs were obtained from the Atak‐S parent flock at 42 weeks of age for the experiment. The experiment was designed in two groups: a control group (C), in which no injection was performed, and the other group in which a solution containing formula products at concentrations of 1.25% (F1), 2.5% (F2) and 5% (F3) was injected into 0.5 mL/egg air sac. The hatching rate, embryonic mortality and discard chick rate were examined at the end of the trial.

**Results:**

The best result of the hatching rate was found in the Group F2, while there was no difference between the control and Group F1 regarding these parameters. The weight and length of the quality chicks were promoted in the Group F1. In Group F3, the injection of the formula product at a rate of 5% had a negative effect on parameters such as hatchability, embryonic mortality, chick length, Pasgar score and yolk sac weight compared to the other groups. Otherwise, in Group F2, there was a significant increase in villus height, crypt depth and lamina muscularis mucosa thickness compared to the other groups (*p *< 0.01).

**Conclusions:**

As a result, it has been concluded that the appropriate rates for the formula product application on chicks are 1.25% and 2.5%, considering the positive effects of the 1.25% and 2.5% rates and the negative effects of the 5% rate.

## Graphical abstract

The current study presents a rich formula as a good alternative to reduce the mortality that occurs during the transfer of chicks to the final coop after hatching. Improvements were observed in the histology of the small intestine and ileum in eggs fed in ovo.

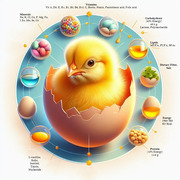

## 1 Introduction

In poultry, due to the natural structure of the egg, it is impossible for external nutrients to enter the egg. All the necessary nutrients for the development of an embryo are obtained within the egg, namely the yolk, albumen and shell. However, the increased metabolic rate during incubation can deplete most of the embryonic nutrient reserves, leading to a deficiency of nutrients before hatching (Yair, Shahar, and Uni 2013). Insufficient nutrients may negatively affect post‐hatch performance and vitality by reducing the nutrient stores of chicks. Therefore, some practices have been developed to prevent nutrient deficiencies, such as adding nutrients to the egg (Şentürk 2019).

The in ovo injection technique, also known as in‐egg feeding, involves injecting nutrients, such as protein, carbohydrates, vitamins and minerals, or substances like hormones and antibodies, into the egg as a liquid solution at any stage of incubation (Herfiana 2007). In ovo feeding was developed by Uni and Ferket (2003) to provide nutrients to the embryo during the incubation period and prevent negative impacts caused by physiological limitations. This biotechnological feeding application is based on the idea that the embryo consumes amniotic fluid close to hatching, and the aim is to transfer the nutrients injected into the amniotic fluid to the intestines (Altan 2018).

1 **Summary**

- To feed the embryo in ovo with rich nutrients
- To development of day‐old chicks fed with formula product
- To affect small intestinal histology of day‐old chicks with formula product

In ovo feeding is a crucial method initiating the development of the intestinal and immune systems of chicks during the embryonic period, prior to hatching. It has been extensively studied and proven to promote chick quality, viability and subsequent performance significantly (Ferket 2011). In addition, in ovo feeding plays a vital role in counteracting the negative effects on hatchability and chick quality that may result from the malnutrition of breeding animals by providing nutrients directly to the eggs (Uni and Ferket 2004). Besides, it increases digestive system capacity, growth rate and feed utilisation while reducing disease and mortality rates after hatching, especially in the first week (Ferket et al. 2005).

Because embryos meet in‐egg nutrient intake after hatching, they easily adapt to exogenous nutrients by consuming less energy. The remaining energy is used for the morphological and physiological development of the gastrointestinal tract (Givisiez et al. 2020). This is very important since optimal nutrient utilisation and animal growth depend on early growth and development of the gastrointestinal tract. Providing early nutrients to the embryos stimulates the intestinal microflora and reduces the delay in early nutrient intake, which can postpone the beneficial stimulation of the immune system. This method has also been supported by recent research conducted by Das, Mishra, and Jha (2021). Intra‐egg helps to combat the negative impacts of starvation in chicks during transfers between hatcheries and production farms, as chicks cannot be fed during this period of approximately 48–72 h.

The most used method to investigate gut health is to evaluate the histomorphology of the gut. The development of the small intestine is related to the digestion and absorption of nutrients. Parameters such as villus height, crypt depth, villus height/crypt depth ratio, enzyme activity and goblet cell number are used to determine intestinal capacity (de Jong et al. 2017). It is reported that as the villus length increases, the intestinal mucosa becomes more capable of digestion and absorption with the development of differentiation (Jeurissen et al. 2002).

About 90% of the embryo's energy needs are met by yellow lipids, with the remaining 10% coming from carbohydrates and proteins. Because of this high ratio, lipids are the main source of energy for the embryo. However, despite the high lipid content of the yolk sac, it is poor in protein. As a result, the amount of protein required by rapidly growing embryos is insufficient to be met by albumin. In fact, due to the insufficient carbohydrate content of the egg, amino acids are used for glycogen production, which reduces protein synthesis from amino acids. For this reason, it has been reported that adding protein to the egg during incubation is beneficial for the development of the embryo (Sözcü 2018). After hatching, poultry needs carbohydrates to provide energy for muscular activity. As the amount of glycogen in the liver and muscles is limited and depleted in a short time, it is stated that adding glycogen reduces the need to produce glucose from amino acids by gluconeogenesis (Uni et al. 2005).

The formula used in this study was selected for its high energy and nutrient content. Commercially available formula products contain 42% carbohydrates, 13% protein and 45% lipids and provide a high nutritional value and many vitamins and minerals necessary for development. This study aimed to determine the effect of in ovo feeding of the formula product on hatching parameters, some organ characteristics and ileal histology. It is believed that the findings of the study may contribute to the development of such formula products for chicks.

## 2 Materials and Methods

### 2.1 Hatching Procedure and In Ovo Injection

The study was carried out at the Ankara Poultry Research Institute Directorate. In this study, 400 fertilised eggs weighing 57–62 g were obtained from 42‐week‐old ATAK‐S hens of the Ankara Poultry Research Institute Directorate. The selected, weighed and numbered breeder eggs were grouped according to their weights. The study was designed with 4 groups and 4 replicates, and each replicate had 25 eggs according to the randomised experimental design. Eggs were arranged on incubation trays, placed on development trolleys and transferred to hatching machines with a temperature of 37.8°C and 50% relative humidity after fumigation. On Day 16 of incubation, the lamps were checked, and unfertilised and dead embryos were removed from the study. For each group, 60 viable eggs were selected, and the experiment was continued with a total of 240 eggs. On the 18th day of incubation (429–430 h), in ovo injection was performed into the air sac.

The powdered product purchased from a commercial company (Table 1) was added in 1.25, 2.5 and 5 g in 100 mL of styryl distilled water to give 1.25%, 2.5% and 5% solutions, respectively. Before in ovo injection, the eggs were disinfected with 75% ethyl alcohol from the blunt parts. The eggs were then punctured from the blunt parts using a piercing tool. Sterile syringes (2.5 mL) and syringe tips (26G and ½″) were used for injection. The experimental groups were designed as non‐injected control (C) and 0.5 mL/egg injected with a solution containing 1.25% (F1), 2.5% (F2) and 5% (F3) of the formula product. After injection, the application area of the eggs was disinfected with 75% alcohol, and the holes caused by the injection were closed with liquid paraffin. The in ovo injected eggs were placed in plastic hatching boxes according to groups and transferred to hatching machines at 36.5°C–37°C temperature and 65%–75% relative humidity. On the 21st day of incubation (504th h), hatching was checked to ensure that it was completed. All chicks were kept in the hatching machine for 2 h for drying.

**TABLE 1:** Energy and nutrients per 100 g of formula product content.

Contents	Unit	Amounts	Content	Unit	Amounts
**Energy**	kJ/kcal	1960/468	**Vitamins**		
**Protein (13% energy)**	g	15.6	Biotin	mcg	21
**Carbohydrate (42% energy)**	g	49.8	Vitamin C	mg	101
Lactose	g	33.2	**Minerals**	—	—
Polysaccharide	g	12.5	Sodium	mg	415
**Lipid (45% energy)**	g	22.9	Potassium	mg	474
Monounsaturated fatty acids	g	8.5	Chloride	mg	504
Polyunsaturated fatty acids	g	4.8	Calcium	mg	593
Saturated fatty acids	g	9.6	Phosphorus	mg	332
**Dietary fibre**	g	4.7	Magnesium	mg	47
**Salt**	g	1.03	Iron	mg	9.5
**Vitamins**			Zinc	mg	6.5
Vitamin A	mcg‐re	2134	Copper	mcg	474
Vitamin D_3_	mcg	18	Manganese	mcg	59
Vitamin E	mgα‐te	21	Selenium	mcg	27
Vitamin K_1_	mcg	36	Iodine	mcg	148
Vitamin B_1_	mcg	830	**Others**		
Vitamin B_2_	mcg	1186	l‐Carnitine	mg	11
Niacin	mg‐ne	19	Kolin	mg	101
Pantothenic acid	mcg	5217	İnositol	mg	142
Vitamin B_6_	mcg	711	Taurin	mg	33
Folic acid	mcg	208	Nucleotide	mg	20
Vitamin B_12_	mcg	1.4			

*Note*: The ingredients of the formula: lactose (from milk), skimmed milk, vegetable oils (palm oil, soybean oil, evening primrose oil, monocellular oil [from seafood], coconut oil, sunflower oil), glucose syrup, whey protein concentrate (from milk), galacto‐oligosaccharide (from milk), medium chain triglycerides, animal fats (fish oil, egg lipid), fructo oligosaccharide, tricalcium phosphate, calcium carbonate, trisodium citrate, sodium chloride, magnesium chloride, inositol, l‐ascorbic acid, tripotassium citrate, choline chloride, emulsifier (soy lecithin), DL‐alpha tocopherol acetate, taurine, ferrous sulphate, retinyl acetate, zinc sulphate, nicotinamide, cytidine 5‐monophosphate, l‐carnitine, uridine 5‐monophosphate sodium salt, calcium d‐pantetonate, inosine 5‐monophosphate sodium salt, cholecalciferol, adenosine 5‐monophosphate, copper sulphate, guanosine 5‐monophosphate sodium salt, retinyl palmitate, thiamine hydrochloride, DL‐alpha tocopherol, pyridoxine hydrochloride, riboflavin, phyterolmonoglutamic acid, cyanocobalamin, potassium iodide, manganese sulphate, sodium selenite, phytomenadione, d‐biotin.

### 2.2 Hatching and Chick Quality Parameters

Parameters related to hatching day parameters and chick quality were calculated as described by Kamanlı and Durmuş (2014) and Sahin and Tasdemir (2017). The formulas used are given below.

*Hatchability*: the number of live chicks hatched / the number of fertilised eggs incubated × 100.

*Embryonic mortality*: the number of embryonic deaths / the number of incubated fertilised eggs × 100.

*Rate of discarded chicks*: the number of discarded chicks / the number of hatched chicks × 100.

*Chick weight*: chick weights were recorded individually using a precision balance.

*Chick length*: the length of all hatched chicks was determined by measuring the length between the beak and the middle claw.

*Chick conversion rate*: weight of hatching chick / initial weight of egg × 100.

*Chick quality score*: determined using the Pasgar score method. All chicks hatched from each group were scored for five traits (agility, beak, legs, yellow absorption and belly).

### 2.3 Yellow Sac and Small Intestine Tract Parameters

To note the weight of the yolk sac, small intestine measurements and ileum histology parameters, randomly selected 6 female chicks from each group (in total, 24 female chicks) were killed by cervical dislocation after their weights were recorded individually. The chicks were opened from the abdominal cavity, and the yolk sacs were removed to record the weight of the yolk sac and the chick without the yolk sac. The relative yolk sac weight was determined by dividing the yolk sac weight by the chick weight. The weight of the small intestine was weighed on a precision balance, and the relative weight of the small intestine was determined by proportioning the weight of the small intestine to the weight of the chick. In addition, the total length of the small intestine and the lengths of its sections (duodenum, jejunum and ileum) were measured. The small intestine length/weight ratio was calculated as suggested in previous studies (Deeming 2005; Yalçin, Izzetoğlu, and Aktaş 2013).

### 2.4 Ileum Histological Parameters

Histological analyses of ileum were performed at Kırşehir Ahi Evran University, Faculty of Agriculture, Department of Animal Science. Small intestine samples were taken from the ileum in 10 mm sections and preserved in 10% formalin until histological analysis. Paraffin cassettes were prepared for histological samples from the ileum, and 5 µm sections were taken. Sections were glued to slides and passed through xylene to remove paraffin. The samples were passed through alkane to remove xylene from the tissues. The clean samples were stained with haematoxylin and eosin stain. Tissue samples were examined under a camera light microscope (ZEISS Primo Star, Germany), and villus length, crypt depth and lamina muscularis mucosal thickness were calculated using ZEN 2012 SP2 image processing and analysis software (Filik et al. 2020).

### 2.5 Statistics Analysis

The data obtained from the experiment were subjected to analysis of variance with the general linear model (PROC GLM) procedure regarding the experimental model (Coincidence Plots Experimental Design) using the SAS 1996, Salahi, Khabisi, and Mousavi (2011) package program. The effect of the formula product on all measured parameters (treatment) was determined by linear, quadratic and cubic regression analysis by defining orthogonal polynomial contrast in the same package (Düzgüneş et al. 1987). Duncan's multiple comparison test was used to determine the difference between groups (Genç and Soysal 2018).

## 3 Findings and Discussion

The effects of in ovo injection of a formula product into the eggs of Atak‐S breeders on hatching parameters, chick quality, small intestine development and ileal histology were investigated. No study was found in the literature that was like the formula. Therefore, the literature comparison was made based on the ingredients the formula contains, such as proteins, carbohydrates, vitamins, minerals, etc.

### 3.1 Hatching Parameters

Table 2 presents the results of the effect of in ovo injection of formula product on hatching parameters. The statistical analysis revealed a significant difference between the groups (*p *< 0.01) considering linear, quadratic and cubic effects (*p *< 0.01) in terms of all parameters. The lowest hatching ratio among groups was observed in the Group F3. Based on these findings, it can be concluded that increasing the dose amount may have a negative impact on hatching parameters.

**TABLE 2:** Effect of formula product on incubation parameters.

Parameters (%)	Treatment groups				*p*	SED	Effects		
	C	F1	F2	F3			L	Q	C
Rate of hatchability	95.00^b^	95.00^b^	96.67^a^	80.00^c^	< **0.0001**	0.00	< **0.0001**	< **0.0001**	< **0.0001**
Rate of embryonic mortality	5.00^b^	3.33^c^	3.33^c^	15.00^a^	< **0.0001**	0.00	< **0.0001**	< **0.0001**	< **0.0001**
Rate of discarded chicks	0.00^c^	1.75^b^	0.00^c^	6.25^a^	< **0.0001**	0.00	< **0.0001**	< **0.0001**	< **0.0001**

*Note*: The trial groups: control (C), group that did not receive any injections, and groups that were injected with 0.5 mL/egg of a solution containing formula products at concentrations of 1.25% (F1), 2.5% (F2) and 5% (F3), respectively.

Abbreviations: C, cubic effect; L, linear effect; *p*, probability; Q, quadratic effect; SED, the standard error of a difference between three means.

^a,b,c^ Mean values within the same column with no common superscripts differ significantly (*p *< 0.01).

In ovo feeding has been extensively studied to examine its effects on hatching parameters (Ohta and Kidd 2001; Salahi, Khabisi, and Mousavi 2011; Zhai, Rowe, and Peebles 2011; Salmanzadeh 2012; Coşkun, Çayan, et al. 2014; Kop‐Bozbay et al. 2016; Edwards, Heberle, and Hynd 2016; Altan et al. 2017). These studies have provided strong evidence for the effectiveness of in ovo feeding in improving hatching parameters. Various application sites, including the air sac, amniotic fluid and yellow sac, as well as different application times, such as Day 12, Day 16, Day 17.5 and Day 18 have been explored. The effects of different incubation temperatures and nutrient contents, including amino acids, carbohydrates, vitamins, minerals, hormones and bee products, on hatching parameters have been extensively studied by various researchers.

Our study unequivocally demonstrates that the hatchability rate significantly decreased in the Group F3 compared to the other groups. It is worth noting that there have been varying results on hatching in the past, representing similar results with our study. For instance, Zhai, Rowe, and Peebles (2011) found that in ovo carbohydrate, and Joshua, Valli, and Balakrishnan (2016) found that in ovo nano zinc, copper and selenium injection did not affect hatchability. Similarly, Çalık (2018) found that in ovo inulin and lactulose, Dal Pont et al. (2019) found that in ovo glycerol and Duan et al. (2021) found that in ovo probiotic and symbiotic injection did not affect hatchability. However, Coşkun, Erener, et al. (2014) reported that in ovo DL‐methionine, Salmanzadeh, Shahryar, and Lotfi (2014) reported in ovo butyric acid and Neves et al. (2017) reported in ovo glycerol application decreased hatchability. Retes et al. (2018) examined 17 in ovo carbohydrate trials in their systematic review and found that 64% of hatchability was reduced regardless of the solution. Finally, Ghane et al. (2021) reported that in ovo injection of vitamin C and vitamin E increased the hatching rate. This study confirms the existing literature, as the decrease observed in the Group F3 and the similarity between the other groups are aligned with previous findings.

The Group F3 had higher rates of embryonic mortality and rejected chicks compared to the other groups, which can be caused by increased dose amount. It is worth noting that previous studies have shown that in ovo amino acid (Shafey et al. 2014) and l‐ascorbic acid treatments (Zhang et al. 2018) did not have any significant impact on embryonic mortality. Consistent with the findings of Sözcü and Ak (2020), our study demonstrated a significant difference between groups in embryonic mortality and rejected chick rates following in ovo glutamine injection. Notably, these rates were observed to increase in a dose‐dependent manner.

### 3.2 Chick Quality

Table 3 shows the effects of the formula product on the determined parameters following in ovo injection. The egg weights of the groups were standardised (*p *> 0.05). The results of chick weight analysis represented no statistically significant difference between the groups (*p *> 0.05). However, the formula product had a significant impact on chick length, with the Group F1 showing the highest length (*p *< 0.01). The Pasgar score calculation results showed a statistically significant difference (*p *< 0.01) between the groups. In addition, the analysis of the chick conversion rate, determined by the ratio of chick hatching weight to egg starting weight, revealed a statistically significant difference (*p *< 0.01) between the groups. The product of the formula had significant effects on chick weight (cubic, *p *< 0.05), chick length (linear and quadratic, *p *< 0.01), Pasgar score (linear and quadratic, *p *< 0.01) and egg/chick ratio (linear, quadratic and cubic, *p *< 0.01).

**TABLE 3:** Effect of formula product on chick quality.

Parameters	Treatment groups				*p*	SED	Effects		
	C	F2	F3	F3			L	Q	C
Egg weight (g)	58.87	58.87	58.88	58.87	1.0000	0.09	0.9995	0.9711	0.9691
Chick weight (g)	40.05^b^	40.94^a^	40.34^ab^	40.67^ab^	0.0841	0.13	0.3041	0.2870	**0.0360**
Chick length (cm)	16.82^b^	17.07^a^	16.84^b^	16.44^c^	< **0**.**0001**	0.04	< **0**.**0001**	< **0**.**0001**	0.3294
Pasgar score	9.84^a^	9.79^a^	9.66^a^	9.25^b^	< **0**.**0001**	0.03	< **0**.**0001**	**0.0106**	0.5283
Egg/chick ratio	67.94^d^	69.55^a^	68.48^c^	69.05^b^	< **0**.**0001**	0.00	< **0**.**0001**	< **0**.**0001**	< **0**.**0001**

*Note*: The trial groups: control (C), group that did not receive any injections, and groups that were injected with 0.5 mL/egg of a solution containing formula products at concentrations of 1.25% (F1), 2.5% (F2) and 5% (F3), respectively.

Abbreviations: C, cubic effect; L, linear effect; *p*, probability; Q, quadratic effect; SED, the standard error of a difference between three means.

^a,b,c^ Mean values within the same column with no common superscripts differ significantly (*p *< 0.01).

Egg weight was reported to have a positive relationship with chick hatching weight (Ulmer‐Franco, Fasenko, and O'Dea Christopher 2010). For this reason, egg weights were adjusted to be similar between the groups in order not to affect chick hatching weights from egg weight. The studies conducted on the impact of chick hatching weight, including Tako, Ferket, and Uni (2004) in the ovo carbohydrate mixture, Uni et al. (2005) in the ovo carbohydrate mixture, Foye, Uni, and Ferket (2006) in the ovo protein and carbohydrate, Salmanzadeh (2012) in the ovo glucose, Shafey et al. (2014) in the ovo amino acids and Kim et al. (2020) in the ovo epidermal growth factor, have found similar results with our study, which is that chick weight was higher in the treatment groups compared to the control group. Oliveira et al. (2015) in ovo Zn, Mn and Cu, Joshua, Valli, and Balakrishnan (2016) in ovo nano mineral substance, Neves et al. (2017) in ovo glycerol, Ebrahimi et al. (2017) in ovo l‐lysine, Dinçgörür Yılmaz and Çelik (2020) in ovo vitamin E and vitamin C, Şentürk and Yıldız (2020) in ovo organic Zn, Cu and Mn, and Cöner and Saçaklı (2023) in ovo glucose and glutamine treatment did not have a significant effect on chick hatching weight, and it was seen that it was in accordance with the results of our current study.

Like chick weight, chick length was also the highest in the F1 group. The results of the control and F2 groups were similar, and the shortest chick length was measured in the F3 group. In different in ovo injection studies, Salahi, Mousavi, et al. (2011) determined chick length in butyric acid treatment, Sözcü (2018) in glutamine treatment, Araújo et al. (2019) in vitamin E treatment and Şentürk and Yıldız (2020) in vitamin E and vitamin C treatment and reported statistically significant differences between treatment groups compared to the control group. The reviewed literature has similar results with our study.

The Pasgar score findings for chick quality indicate that all groups of chicks scored above 9 points, demonstrating good quality. The control group achieved the highest score, while the Group F3 obtained the lowest score and lagged the other groups in hatching parameters. Based on these results, it can be confidently predicted that an increase in formula product doses may have negative effects. Our current results are consistent with Sözcü's (2018) study, which found that the highest dose group had the lowest Tona score. These findings support our study's conclusions with confidence. Similarly, previous studies by Shafey et al. (2012), Shafey et al. (2014), Coşkun Erener, et al. (2014) and Sözcü and Ak (2020) have all reported an increase in the proportional chick conversion rate with in ovo carbohydrate, amino acid, DL‐methionine and glutamine injection, respectively.

### 3.3 Yellow Sac and Small Intestine Tract Parameters

The data obtained from the application of the formula product indicate that the treatments did not have a significant effect on chick weight, yolk sac weight and chick weight without yolk sac (*p *> 0.05). However, a linear effect (*p *< 0.05) was observed between the groups only in the yolk sac weight parameter. The relative yolk sac ratio showed a statistically significant difference and a linear effect (*p *< 0.05).

Chicks pull the yolk sac into their abdominal cavity before hatching. To obtain accurate results, it is important to consider the weight of the yolk sac, which is added to the chick's weight. Therefore, measuring only the weight of the chicks may not be sufficient. To calculate the average chick weight more accurately, it is recommended to weigh the yolk sacs of a sample of chicks of a certain size from a batch of hatches (Şeremet 2012). Table 4 presents the results of the chick weight, yolk sac weight and chick weight without the yolk sac. The weight of the chick without the yolk sac may not have been statistically significant, but the differences of more than 1 g between the groups are noteworthy. This underscores the significance of measuring the yolk sac to determine the actual weight of the chick. The relative weight of the yolk sac increased in parallel with the dose, indicating that in ovo application reduced yolk sac utilisation with increasing dose. Şeremet (2012) stated that a high yolk sac weight indicates that embryos were unable to effectively utilise the yolk sac for development during the incubation period. The present study's results confirm this finding, as the yolk sac weight increased with increasing dose rate, while the weight of chicks without yolk sac decreased. The increase in dose rate restricted the use of the yolk sac, resulting in negative effects on the embryo's development and a decrease in the weight of the chick without a yolk sac. Wolanski et al. (2007) found significant effects on inter‐chick yolk sac weight and relative yolk sac difference. However, Bello et al. (2013) reported no significant effect on yolk sac free chick weight with in ovo calcidiol administration. Similarly, Maman et al. (2019) found no significant effect on chick weight, yolk sac‐free chick weight and relative yolk sac weight with in ovo vitamin D3 injection. Cöner and Saçaklı (2023) also reported no significant effect on yolk sac weight with in ovo glucose, glutamine and glucose + glutamine. According to Şentürk and Yıldız's (2020) findings, the weight of the yolk sac was increased through the application of organic Zn, Cu and Mn in ovo. The study results indicate that the administration of the formula product did not significantly affect small intestine measurements (*p *> 0.05). These findings are supported by the data presented in Table 5. However, a linear effect (*p *< 0.05) was observed on ileum length.

**TABLE 4:** Effect of formula product on yellow sac.

Parameters	Treatment groups				*p*	SED	Effects		
	C	F1	F2	F3			L	Q	C
Chick weight (g)	39.23	39.56	38.67	39.15	0.928	0.47	0.7937	0.9347	0.5463
Yellow sac weight (g)	4.67^ab^	4.49^b^	5.15^ab^	6.11^a^	0.135	0.25	**0.0388**	0.2681	0.8097
Unhatched chick weight (g)	34.56	35.07	33.52	33.04	0.184	0.35	0.0651	0.4870	0.3260
Relative yellow sac (%)	11.90^b^	11.24^b^	13.23a^b^	15.54^a^	**0.048**	0.53	**0.0138**	0.1806	0.6310

*Note*: The trial groups: control (C), group that did not receive any injections, and groups that were injected with 0.5 mL/egg of a solution containing formula products at concentrations of 1.25% (F1), 2.5% (F2) and 5% (F3), respectively.

Abbreviations: C, cubic effect; L, linear effect; *p*, probability; Q, quadratic effect; SED, the standard error of a difference between three means.

^a,b,c^ Mean values within the same column with no common superscripts differ significantly (*p *< 0.01).

**TABLE 5:** Effect of formula product on small intestine measurements.

Parameters	Treatment groups				*p*	SED	Effects		
	C	F1	F2	F3			L	Q	C
Small intestine weight (g)	0.98	0.91	0.96	0.87	0.667	0.04	0.3637	0.9061	0.4087
Small intestine length (cm)	30.06	30.51	32.22	31.43	0.562	0.58	0.2712	0.5958	0.4728
Duodenum length (cm)	6.90	6.33	6.43	6.39	0.670	0.18	0.3857	0.4715	0.6229
Jejunum length (cm)	13.04	13.27	13.73	13.04	0.743	0.25	0.8367	0.3750	0.5429
Ileum length (cm)	10.12^b^	10.92^ab^	12.07^a^	12.00^a^	0.061	0.28	**0.0120**	0.4419	0.5345
Small intestine relative weight (%)	2.51	2.31	2.50	2.24	0.682	0.10	0.4805	0.8676	0.3353
Small intestine weight/length	3.27	2.96	2.97	2.76	0.221	0.08	0.0536	0.7830	0.4833

*Note*: The trial groups: control (C), group that did not receive any injections, and groups that were injected with 0.5 mL/egg of a solution containing formula products at concentrations of 1.25% (F1), 2.5% (F2) and 5% (F3), respectively.

Abbreviations: C, cubic effect; L, linear effect; *p*, probability; Q, quadratic effect; SED, the standard error of a difference between three means.

^a,b,c^ Mean values within the same column with no common superscripts differ significantly (*p *< 0.01).

Table 5 presents the results of length and weight measurements of small intestine sections. The data clearly showed that there was no statistically significant difference between the groups in any of the parameters, except for ileum length, which exhibited a linear effect. Our study is consistent with Salahi, Khabisi, and Mousavi (2011) finding that in ovo injection of butyric acid did not affect the length of the duodenum and ileum on the day of hatching. However, we found a significant difference in the length of the jejunum and total small intestine, which contradicts their findings. The results of small intestine weight were similar to our study. Our study differs from X. Y. Zhang et al. (2018) in that we did not find a significant difference in the relative weight of the small intestine between the groups. However, Ebrahimi et al. (2017) did find an effect on the lengths of the duodenum, jejunum and ileum with in ovo injection of l‐lysine. The study found no significant difference in the lengths of the duodenum and ileum but did find a statistically significant difference in the length of the jejunum between the groups. Our study produced similar results for the duodenum and ileum but demonstrated a statistically significant difference for the jejunum.

### 3.4 Ileum Histological Parameters

The study confidently concludes that formula product injection has a significant impact on ileum histology in chicks, as evidenced by the statistical analysis of Table 6. The results show significant differences in villus length, crypt depth, lamina muscularis mucosa thickness and villus length/crypt depth (VL/CD) groups (*p *< 0.01). Villus length, crypt depth, lamina muscularis mucosa thickness and the VL/CD ratio all demonstrated significant effects (*p *< 0.01). Villus length exhibited a cubic effect, while crypt depth showed both linear and quadratic effects. Lamina muscularis mucosa thickness demonstrated a cubic effect, and the VB/CD ratio showed both quadratic and cubic effects.

**TABLE 6:** Effect of formula product on histology of ileum.

Parameters (µm)	Treatment groups				*p*	SED	Effects		
	C	F1	F2	F3			L	Q	C
Villus length	288.51^b^	281.04^b^	304.03^a^	288.01^b^	< **0.0001**	1.43	0.0870	0.1362	< **0.0001**
Crypt depth	35.62^c^	37.95^a^	38.29a	36.91^b^	< **0.0001**	0.18	**0.0073**	< **0.0001**	0.8686
Lamina muscularis mucosa	93.68^ab^	86.54^c^	96.13^a^	90.81^b^	< **0.0001**	0.66	0.8641	0.4908	< **0.0001**
VL/CD ratio	8.29^a^	7.55^c^	8.12^ab^	7.94^b^	< **0.0001**	0.05	0.2788	**0.0062**	< **0.0001**

*Note*: The trial groups: control (C), group that did not receive any injections, and groups that were injected with 0.5 mL/egg of a solution containing formula products at concentrations of 1.25% (F1), 2.5% (F2) and 5% (F3), respectively.

Abbreviations: C, cubic effect; L, linear effect; *p*, probability; Q, quadratic effect; SED, the standard error of a difference between three means.

^a,b,c^ Mean values within the same column with no common superscripts differ significantly (*p *< 0.01).

During the final stage of incubation, it has been observed that the weight of the intestine experiences the greatest increase in comparison to the weight of the embryo. According to Uni, Tako, et al. (2003), the ratio of intestinal weight to body weight was 1% in 17‐day‐old embryos and 3.5% on the day of hatching. Although intestinal development begins during the embryonic period, it is widely acknowledged that this process accelerates significantly as chicks receive nutrients. Therefore, early feeding is crucial, as highlighted by Givisiez et al. (2020). The final stage of incubation brings about a rapid change that increases the energy requirement and reduces the nutrients available to the embryo within the egg (De Oliveira, Uni, and Ferket 2008). It is believed that in ovo feeding during this stage can positively impact intestinal development and prevent potential problems resulting from nutrient deficiency between the final embryonic period and the chick's introduction to feed (Wang et al. 2020).

After a thorough examination of the results presented in Table 6, it was observed that villus length, crypt depth, lamina muscularis mucosa thicknes*s* and VL/CD ratios were statistically significant (*p *< 0.01) in all groups. Our analysis of various in ovo studies in the literature has shown that there have been numerous investigations into the effects of different nutrients on small intestinal histology. According to Tako, Ferket, and Uni (2004), the in ovo use of a carbohydrate mixture and hydroxy methyl butyrate was studied. Smirnov et al. (2006) found that in ovo use of carbohydrates resulted in increased villus length and surface area, both of which are parameters of small intestinal histology. Cheled‐Shoval et al. (2011) reported that in ovo administration of mannan oligosaccharide (MOS) increased villus length, villus area, crypt depth, goblet cell number and mucosa thickness in the jejunum. In their 2016 study, Bogucka et al. (2016) investigated the impact of in ovo symbiotic and prebiotic treatment on the histology of the ileum of broiler chicks after 1 day. The study found that, while the Symbiotic 2 group had a lower villus length compared to the control group, the other treatment groups showed an increase in villus length. Berrocoso et al. (2017) reported that on the day of emergence of in ovo raffinose injection, the villus length, crypt depth and VL/CD parameters of ileum histology in broiler chicks were statistically significant. Neves et al. (2017) examined the effects of in ovo glycerol administration on ileum histology and reported that increasing doses resulted in increased villus length and crypt depth compared to the control group. Nazem et al. (2017) investigated the effect of in ovo methionine administration on ileum histology and found that it increased villus height. Sözcü and Ak (2020) measured the parameters of the ileum histology of broiler chicks on the day of emergence of in ovo glutamine administration. They reported that villus length and crypt depth increased, whereas VL/CD ratio and lamina muscularis mucosa thickness remained unchanged. Our study produced similar results to the literature cited. Based on the results obtained, it is suggested that the application of in ovo formula may lead to an improvement in the histological characteristics of the ileum. This improvement could potentially contribute to the production of healthy chicks with high viability.

## 4 Conclusion

The study found that the properties examined varied depending on the formula product ratios used. Solutions containing formula products at concentrations of F1 (1.25%), F2 (2.5%) and F3 (5%) were used in the study. Specifically, Group F3 received a 5% formula product dosage, which can be considered as high illustrated negative effects on the examined parameters compared to the other groups. Therefore, it may be advisable for future studies to avoid exceeding a 5% dosage. When considering F1 (1.25%) and F2 (2.5%) groups in this study, it was observed that the formula product administered via in ovo injection did not have any negative effects and even had positive effects on many parameters compared to the control group. In addition, F1 (1.25%) and F2 (2.5%) groups did not show any negative effects compared to the control group on the hatching performance. The study also found that Group F1 (1.25%) demonstrated an improvement in chick quality, specifically in chick weight, yellow sac weight and chick length. Furthermore, Group F2 (2.5%) exhibited longer villus length, crypt depth and lamina muscularis mucosa thickness than all other groups. These results suggest that both F1 (1.25%) and F2 (2.5%) have positive effects on chick quality and ileum histology, respectively. It has been concluded that the in ovo application of the formula product to breeder chicken eggs on the 18th day of incubation through the air sac is feasible and has positive effects on chicks. However, to obtain more detailed results, further studies should be conducted and added to the literature.

## Author Contributions

Oğuzhan Eray and Gökhan Filik designed and conducted the experiments. Oğuzhan Eray performed laboratory analyses. Gökhan Filik supervised and coordinated the experiments. Oğuzhan Eray and Gökhan Filik evaluated experimental data statistically. The manuscript was written and revised by Oğuzhan Eray and Gökhan Filik.

## Ethics Statement

The approval of the Local Ethics Committee for Animal Experiments was obtained by decision of the Local Ethics Committee for Animal Experiments of the Directorate of the Ankara Poultry Research Institute of the Ministry of Agriculture and Forestry dated 12/03/2021 and 2021/02.

## Conflicts of Interest

The authors declare no conflicts of interest.

## Data Availability

The original contributions presented in the study can be directed to the corresponding author.
